# Does *COMT* Play a Role in Parkinson’s Disease Susceptibility Across Diverse Ancestral Populations?

**DOI:** 10.1101/2025.04.11.25325572

**Published:** 2025-04-11

**Authors:** Miguel Martín-Bórnez, Nisar Shar, Mohamed Ahmed Nour, David Murphy, Inas Elsayed, Shri N Megha, Francisca Nwaokorie, Adedunni Olusanya, Nicole Kuznetsov, Sara Bandres-Ciga, Alastair J Noyce, Hirotaka Iwaki, Lietsel Jones, Pilar Gómez-Garre, Pablo Mir, Maria Teresa Periñan

**Affiliations:** 1 Unidad de Trastornos del Movimiento, Servicio de Neurología, Instituto de Biomedicina de Sevilla, Hospital Universitario Virgen del Rocío/CSIC/Universidad de Sevilla, Seville, Spain; 2 Centro de Investigación Biomédica en Red sobre Enfermedades Neurodegenerativas, Instituto de Salud Carlos III, Madrid, Spain; 3 Department of clinical and Movement Neurosciences, UCL Queen Square Institute of Neurology, London WC1N 3BG, UK; 4 NED University of Engineering & Technology, Karachi, Pakistan; 5 UCL Queen Square Institute of Neurology, Faculty of Brain Sciences, UK; 6 Faculty of Pharmacy, University of Gezira, Wadmedani, Sudan; 7 Systems Biology Ireland, School of Medicine and Medical Science, University College Dublin, Dublin, Ireland.; 8 Department of Neurology, National Institute of Mental Health and Neurosciences, India; 9 Department of Medical Laboratory Science, Faculty of Basic Medical Sciences, College of Medicine, University of Lagos, Nigeria; 10 Department of Pharmacology, Therapeutics and Toxicology, Faculty of Basic Medical Sciences, College of Medicine, University of Lagos, Nigeria; 11 Center for Alzheimer’s and Related Dementias (CARD), National Institute on Aging and National Institute of Neurological Disorders and Stroke, National Institutes of Health, Bethesda, MD, USA; 12 Data Tecnica, Washington DC, USA; 13 Center for Preventive Neurology, Wolfson Institute of Population Health, Faculty of Medicine and Dentistry, Queen Mary University of London, London, UK; 14 Departamento de Medicina, Facultad de Medicina, Universidad de Sevilla, Seville, Spain

**Keywords:** Parkinson’s disease, genetics, COMT, dyskinesia, cognitive impairment

## Abstract

**Background:**

The catechol-O-methyltransferase (*COMT*) gene is involved in brain catecholamine metabolism, but its association with Parkinson’s disease (PD) risk remains unclear.

**Objective:**

To investigate the relationship between *COMT* genetic variants and PD risk across diverse ancestries.

**Methods:**

We analyzed *COMT* variants in 2,251 PD patients and 2,835 controls of European descent using whole-genome sequencing from the Accelerating Medicines Partnership-Parkinson Disease (AMP-PD), along with 20,427 PD patients and 11,837 controls from 10 ancestries using genotyping data from the Global Parkinson’s Genetics Program (GP2).

**Results:**

Utilizing the largest case-control datasets to date, no significant enrichment of *COMT* risk alleles in PD patients was observed across any ancestry group after correcting for multiple testing. Among Europeans, no correlations with cognitive decline, motor function, motor complications, or time to LID onset were observed.

**Conclusions:**

These findings emphasize the need for larger, diverse cohorts to confirm the role of *COMT* in PD development and progression.

## Introduction

Parkinson’s disease (PD) is a progressive neurodegenerative disorder marked by motor symptoms such as tremors, rigidity, and bradykinesia, alongside diverse non-motor manifestations. Its etiology involves a complex interplay of genetic and environmental factors [1,2].

The enzyme catechol-O-methyltransferase (COMT; OMIM:116790) plays a crucial role in catecholamine metabolism in the brain, including dopamine and norepinephrine. *COMT* variants can affect enzyme activity and dopamine metabolism [3]. While numerous studies have explored associations between *COMT* variants and PD susceptibility [4], particularly the Val158Met (rs4680), and their impact on levodopa-induced dyskinesia (LID) [5,6], cognitive decline [7–9], and motor fluctuations [10], findings remain inconsistent, leaving the role of *COMT* and variants in close proximity unresolved. Noteworthy, *COMT* is located within the 1.5 Mb region deleted in 22q11.2 deletion syndrome, a genetic disorder caused by the deletion of a small chromosome 22 segment, typically resulting in a wide variety of symptoms, including neurological [11].

We comprehensively investigated whether genetic variation within *COMT* influences PD risk, LID, cognitive impairment, motor function, and complications, utilizing large-scale array-based genotyping and whole-genome sequencing (WGS) data from the Global Parkinson’s Genetics Program (GP2) [12] and the Accelerating Medicines Partnership - Parkinson Disease (AMP-PD) initiatives.

## Methods

We analyzed AMP-PD WGS data release 3.0 (https://amp-pd.org/), including 2,251 unrelated PD patients and 2,835 controls of European descent (Supplementary Table 1). Additionally, we utilized large-scale genotyping imputed data from GP2 release 7 (https://gp2.org/), comprising 20,427 PD patients and 11,837 controls from ten ancestry populations: European (EUR), African Admixed (AAC), African (AFR), Ashkenazi Jews (AJ), American Admixed (AMR), Central Asian (CAS), East Asian (EAS), Middle Eastern (MDE), South Asian (SAS), and Complex Admixture History (CAH).

The GenoTools pipeline (https://github.com/GP2code/GenoTools) was used for genetic ancestry prediction, and quality control (QC) on genotyping data was conducted according to previously described methods [13]. Variants with a Hardy-Weinberg Equilibrium (HWE) p-value≤1×10^−4^ in control samples were removed after preliminary QC. Variants were further pruned to exclude those with a minor allele frequency (MAF)≤1% and a minor allele count of 2. *COMT* gene positions were obtained from Ensembl (https://www.ensembl.org). Variants were annotated using ANNOVAR [14]. Gene-based burden analysis was performed to assess the cumulative effect of potentially functional (variants annotated as frameshift, nonframeshift, startloss, stoploss, stopgain, splicing, missense, exonic, untranslated region at the 5’ end (UTR5), untranslated region at the 3’ end (UTR3), upstream [−100bp], downstream [+100bp], or ncRNA), coding (variants annotated as frameshift, nonframeshift, startloss, stoploss, stopgain, splicing, or missense) and loss-of-function (variants annotated as frameshift, startloss, stopgain, or splicing) variants on PD risk using RVTESTS [15]. SNP-phenotype association analyses were performed with a generalized linear model in PLINK 2.0 [16]. In the logistic regression analysis, we included sex, age, and the first five genetic principal components (PCs) as covariates to account for population stratification. The Bonferroni correction was applied to adjust the p-values, accounting for all *COMT* variants within each ancestry independently. Power calculations were performed using the online Genetic Association Study (GAS) Power Calculator (https://csg.sph.umich.edu/abecasis/cats/gas_power_calculator/index.html).

We examined clinical data of individuals of European ancestry in GP2 and AMP-PD to explore the impact of *COMT* variants on cognitive and motor function, as well as LID development. Cognitive decline was assessed using MoCA scores (N for GP2=568; N for AMP-PD=1341) and linear regression models, adjusting for age, sex, education level, and PCs. We evaluated associations with MDS-UPDRS Parts III (N for GP2=1268; N for AMP-PD=1666) and IV (N for GP2=359; N for AMP-PD=1794) using the same covariates. LID associations were assessed using Cox proportional hazards models, incorporating time-to-event data for LID onset and adjusting for relevant covariates (age, sex, PCs, and levodopa equivalent daily dose). Kaplan-Meier survival curves were used to visualize time-to-LID onset across *COMT* variants.

## Results

Leveraging the AMP-PD WGS data, we identified 491 variants within the *COMT* gene region, including 444 intronic, 13 exonic (8 synonymous, 4 missense, and 1 nonframeshift (3-bp) deletion), and 34 UTR variants (Supplementary Table 2). Association tests for PD risk revealed no significant results for any *COMT* variants (Supplementary Table 3).

**Figure 1.**
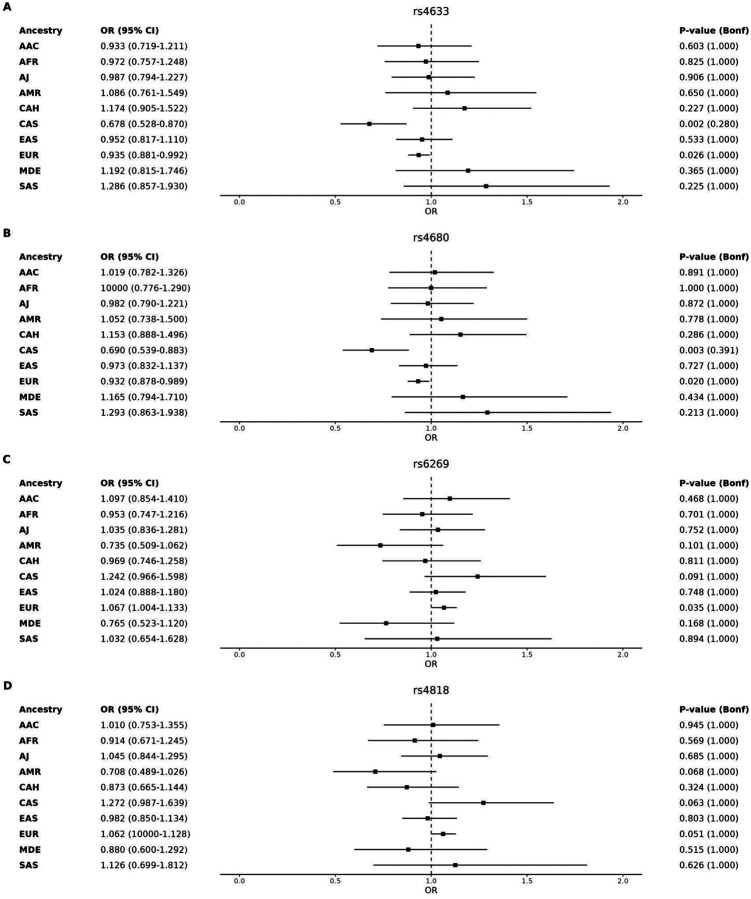
Forest plot illustrating the association between Parkinson’s disease risk and the four most extensively studied *COMT* variants (rs4633, rs4680, rs6269, rs4818) across ten ancestries. The analysis was performed using a generalized linear model adjusted for age, sex, and five principal components. **(A)** rs4633, **(B)** rs4680, **(C)** rs6269, and **(D)** rs4818. Abbreviations: AAC, African Admixed; AFR, African; AMR, American Admixed; Bonf, bonferroni; CAS, Central Asian; CI, confidence intervals; EAS, East Asian; EUR, European; MDE, Middle Eastern; OR, odds ratio; SAS, South Asian.

For the GP2 genotyping imputed data, all identified variants are listed in Supplementary Table 2. In the GP2 CAS ancestry group, nominally significant associations with PD risk were observed for p.Val158Met (rs4680) (Effect Allele=A; Odds Ratio [OR]=0.690, 95% Confidence Interval [CI]=0.539–0.883, p=0.003, Bonferroni-corrected p=0.391) and p.His62=(rs4633) (Effect Allele = T; OR=0.678, 95% CI=0.528–0.870, p=0.002, Bonferroni-corrected p=0.280) (Figure 1, Supplementary Table 4). In the GP2 EUR ancestry group, p.Val158Met (Effect Allele=A; OR=0.932, 95% CI=0.878–0.989, p=0.020, Bonferroni-corrected p=1), p.His62=(Effect Allele=T; OR=0.935, 95% CI=0.881–0.992, p=0.026, Bonferroni-corrected p=1), and c.−98A>G (rs6269) (Effect Allele=G; OR=1.067, 95% CI=1.067–1.133, p=0.035, Bonferroni-corrected p=1) were nominally significantly associated with PD risk. Additionally, the c.*764C>T (rs165728) variant was nominally significantly associated with PD risk in the GP2 AJ (Effect Allele=C; OR=0.602, 95% CI=0.373–0.971, p=0.038, Bonferroni-corrected p=1) and MDE (Effect Allele=C; OR=2.475, 95% CI=1.03–5.949, p=0.043, Bonferroni-corrected p=1) ancestry groups. Finally, p.Ala72Ser (rs6267) was found to be nominally significantly associated with PD risk in the EAS ancestry group (Effect Allele=T; OR=0.643, 95% CI=0.415–0.998, p=0.049, Bonferroni-corrected p=1). However, these associations did not remain significant after multiple testing corrections. Analysis of the latest PD risk GWAS summary statistics [17–21] did not identify significant associations (p-value threshold of 5×10^−8^) with *COMT* genetic variation (Supplementary Figure 1).

Gene-based burden analysis evaluated the cumulative effects of specific *COMT* variant sets on PD risk. In the GP2 AJ ancestry group, coding variants showed a nominal significant association with PD risk (N=3; SKAT p=0.069, SKAT-O p=0.036). Similarly, in the GP2 EUR ancestry group, coding variants (N=18; SKAT p=0.020, SKAT-O p=0.019) but also potentially functional variants were significantly associated with PD risk (N=105; SKAT p=0.032, SKAT-O p=0.037). However, none of these associations remained significant after multiple testing corrections and therefore these results should be interpreted with caution (Supplementary Table 5). In AMP-PD, potentially functional variants showed suggestive nominal evidence of association (N=46; SKAT p=0.071, SKAT-O p=0.110). Since loss-of-function variants were absent in all the groups examined, our study did not perform a burden analysis on them.

Regarding the role of *COMT* in influencing cognitive, motor function and complications in PD, both p.Val158Met (Effect Allele=G; BETA=−0.338; SE=0.150, p=0.024, Bonferroni-corrected p=1) and p.His62=(Effect Allele=C; BETA=−0.330; SE=0.149, p=0.027, Bonferroni-corrected p=1) showed nominal associations with MoCA scores in the GP2 EUR ancestry group. Similarly, these two variants were nominally associated with MDS-UPDRS Part IV scores in the AMP-PD WGS data, along with c.*764C>T (Effect Allele=T; BETA=0.666; SE=0.320, p=0.038, Bonferroni-corrected p=1). However, none of these associations remained significant after correction for multiple testing (Supplementary Tables 6–9).

The risk of developing LID was evaluated in 463 European PD patients from GP2, 34.3% of whom developed LID. The analysis focused on four *COMT* variants (rs6269, rs4633, rs4818, and rs4680) based on their functional significance, as they collectively modulate COMT activity [22]. No significant associations with time-to-LID onset were found in the Cox proportional hazards model (p-value>0.05). Kaplan-Meier survival curves are presented in Supplementary Figure 2.

## Discussion

This study leveraged AMP-PD WGS and GP2 genotyping data, representing the largest and most ancestrally diverse dataset to date, spanning ten different ancestries. It provides a unique and comprehensive analysis of *COMT* variants and their impact on PD risk across populations. Recent work by Poplawska et al. (2024) emphasizes the lack of ethnic diversity in clinical trials involving COMT inhibitors, underscoring the importance of our study in addressing the inclusion of underrepresented populations in research on *COMT* variants and PD risk [23].

No statistically significant associations were found for any ancestral populations in our analysis, including EAS, SAS and EUR, which have been the primary focus of previous research [4,9,24,25]. Notably, significant associations have been reported in Asian populations, particularly Japanese and Chinese cohorts (EAS), and to a lesser extent in Indian cohorts (SAS) [24–26]. A study involving 109 Japanese PD patients and 153 controls found a significant association with the homozygous p.Val158Met genotype over two decades ago [27]. This association was further supported by a meta-analysis of Asian populations, including 1,581 PD patients and 1,376 controls [26]. Despite having adequate statistical power (80% power to detect an OR≥1.5 for a MAF≥10%), our analysis of a large EAS cohort, consisting of 2,646 PD patients and 2,453 controls, did not replicate these findings. Similarly, in SAS populations, a meta-analysis of 489 Indian PD patients and 823 controls found a significant association between p.Val158Met and PD risk [25], but our study was unable to replicate this result. Future research should focus on increasing sample sizes in these populations to improve robustness.

Our study is the largest to date investigating p.Val158Met in European ancestry. A previous meta-analysis of 9,719 PD patients and 14,634 controls found no significant association between this variant and PD risk in European populations [4]. Another meta-analysis of 11,428 PD cases and 16,726 controls also reported no significant associations for p.Val158Met in either European or Asian ancestries [9]. Our results are consistent with these findings, further supporting the lack of association with sufficient statistical power.

Levodopa-induced dyskinesia (LID) is a major complication of long-term levodopa therapy in PD, affecting 20%−40% of patients [28]. In our study, 34.3% of patients developed LID, all of whom were on levodopa treatment. While we found no significant associations between *COMT* variants and LID in European ancestry, our findings align with a recent meta-analysis, which also reported no genome-wide significant associations between *COMT* variants and time-to-LID [28]. However, previous studies have suggested that the rs4680 genotype may modulate LID risk in PD patients from European [29], Asian and Brazilian ancestries [5,6,10]. We also examined the relationship between *COMT* variants and motor function, as assessed by MDS-UPDRS Parts III (motor function) and IV (motor complications, including LID). We found no significant associations with motor function or complications in the GP2 EUR ancestry group after correction for multiple comparisons. A meta-analysis of 1,574 Asian PD patients reported a significant association between rs4680 and higher UPDRS III scores [9], but we did not replicate this in Europeans. This may reflect population-specific genetic architecture or limited power for smaller effect sizes.

Similarly, we found no significant association with *COMT* variants and cognitive decline in the GP2 EUR ancestry group. This aligns with results from a meta-analysis of p.Val158Met, which also reported no significant link to cognitive decline in PD [9]. However, a longitudinal study involving 246 PD patients suggested that homozygous carriers of rs4680 may experience accelerated cognitive decline [30].

In conclusion, our results show no association between *COMT* and PD risk that passes multiple test correction, emphasizing the need for larger, diverse cohorts to confirm its role in PD development and progression.

## Supplementary Material

Supplement 1

## Data Availability

Data used in the preparation of this article were obtained from the Global Parkinson’s Genetics Program (GP2; https://gp2.org). Specifically, we used Tier 2 data from GP2 release 7 (DOI:10.5281/zenodo.10962119). Tier 1 data can be accessed by completing a form on the Accelerating Medicines Partnership in Parkinson’s Disease (AMP^®^-PD) website (https://amp-pd.org/register-for-amp-pd). Tier 2 data access requires approval and a Data Use Agreement signed by your institution. All code generated for this article, and the identifiers for all software programs and packages used, are available on GitHub [https://github.com/GP2code/COMT-PD-GeneAnalysis] and were given a persistent identifier via Zenodo [DOI 10.5281/zenodo.15185052].
